# Using 454 technology for long-PCR based sequencing of the complete mitochondrial genome from single *Haemonchus contortus *(Nematoda)

**DOI:** 10.1186/1471-2164-9-11

**Published:** 2008-01-11

**Authors:** Aaron R Jex, Min Hu, D Timothy J Littlewood, Andrea Waeschenbach, Robin B Gasser

**Affiliations:** 1 Department of Veterinary Science, The University of Melbourne, 250 Princes Highway, Werribee, Victoria, 3030, Australia; 2 Department of Zoology, The Natural History Museum, Cromwell Road, London, UK

## Abstract

**Background:**

Mitochondrial (mt) genomes represent a rich source of molecular markers for a range of applications, including population genetics, systematics, epidemiology and ecology. In the present study, we used 454 technology (or the GS20, massively parallel picolitre reactor platform) to determine the complete mt genome of *Haemonchus contortus *(Nematoda: Trichostrongylidae), a parasite of substantial agricultural, veterinary and economic significance. We validate this approach by comparison with mt sequences from publicly available expressed sequence tag (EST) and genomic survey sequence (GSS) data sets.

**Results:**

The complete mt genome of *Haemonchus contortus *was sequenced directly from long-PCR amplified template utilizing genomic DNA (~20–40 ng) from a single adult male using 454 technology. A single contig was assembled and compared against mt sequences mined from publicly available EST (NemBLAST) and GSS datasets. The comparison demonstrated that the 454 technology platform is reliable for the sequencing of AT-rich mt genomes from nematodes. The mt genome sequenced for *Haemonchus contortus *was 14,055 bp in length and was highly AT-rich (78.1%). In accordance with other chromadorean nematodes studied to date, the mt genome of *H. contortus *contained 36 genes (12 protein coding, 22 tRNAs, *rrnL *and *rrnS*) and was similar in structure, size and gene arrangement to those characterized previously for members of the Strongylida.

**Conclusion:**

The present study demonstrates the utility of 454 technology for the rapid determination of mt genome sequences from tiny amounts of DNA and reveals a wealth of mt genomic data in current databases available for mining. This approach provides a novel platform for high-throughput sequencing of mt genomes from nematodes and other organisms.

## Background

The mitochondrion is the organelle responsible for cellular respiration and energy production in many eukaryotic organisms. In addition to its role in cellular function, the mitochondrion contains an internalised, usually circular genome (~13–20 kb in size) which is separate to, but co-operates with, the nuclear genome [1-4]. Knowledge about mt genomes and their structure provides a basis for investigating intracellular physiology and biochemistry [5,6], and gives insights into mt disorders/diseases caused by mt gene mutations [7,8]. Also, because the mt genome is relatively large, the genome structure is highly conserved, and many of the mt genes are highly variable, genetic markers in mt genomes are useful for taxonomic, ecological, population genetic and evolutionary studies (reviewed by [3,4,9]). However, for some groups of organisms, particularly invertebrates, there is very limited information on mt genomes, which most likely relates to the inaccessibility of a practical, generally applicable and cost-effective technique for mt genome sequencing. In the past, mt genome sequencing relied mainly on the purification of mtDNA from the organism under study and its subsequent cloning (with or without PCR) [10,11], sequencing and sequence assembly to then determine the genome structure and gene order. For vertebrates and large invertebrates, where microgram or milligram amounts of mtDNA can be isolated and purified from individuals, this conventional procedure is effective. However, for small invertebrates, such as tiny parasitic worms (= helminths), the amount of mtDNA which can be purified from individuals is much too small to use this approach. Also, there can be major problems with extensive sequence variation among individuals and/or AT-richness (in some regions of the mt genome) [12,13], preventing accurate sequence determination. An effective long PCR-based method has been established for the amplification and subsequent sequencing from individuals *via *primer walking [14]. However, it has not yet been possible to directly sequence an entire mt genome sequence in a single reaction.

With the recent focus on the sequencing of complete nuclear genomes has come a substantial interest in the development of high-throughput, low cost sequencing platforms capable of much more substantial sequence outputs than has been possible with conventional sequencers. A particularly promising approach is the "massively parallel picolitre reactor platform" (or 454 technology, Life Sciences) [15-21]. The maximum total sequence length which can be determined using this method is presently ~25 Mbp [18], which vastly exceeds the total length of the mt genome. A recent study [22] demonstrated that 454 technology is more reliable than a conventional (Sanger) approach for sequencing highly AT-rich regions, suggesting that this approach could enhance long PCR-based mt genome sequencing. Moreover, the final sequence output obtained *via *the 454 platform can be assembled as one or more contigs from a very large number of short overlapping sequence reads, this platform may offer a more accurate output due to substantial coverage and improved bioinformatic processing following sequencing.

In the present study, we utilized a long-PCR-coupled 454 technological approach to sequence the complete mt genome from a small portion of the genomic DNA from a single adult of *Haemonchus contortus *(Nematoda: Strongylida). This nematode represents a blood-feeding parasite of paramount importance, as a pathogen in small ruminants (sheep and goats), causing anaemia and associated complications, leading to death in severely affected animals [23] and belongs to a group of nematodes (Strongylida) parasitising animals, which cause major disease problems, resulting in substantial economic losses to agricultural and livestock industries worldwide. In order to validate sequencing by this approach, we used mt data for *H. contortus *mined from public databases as a scaffold for mapping and assembly. Also, we characterized the mt genome of this important parasite and compared its genome structure with previously published mt genomes for other strongylid nematodes.

## Results and Discussion

### Molecular verification of species identity, and quality of long-PCR amplicons

A sample of total genomic DNA was isolated from a single adult male of *H. contortus *(McMaster strain) for the sequencing of the complete mt genome. In order to ensure the specific identity of the specimen prior to sequencing, the second internal transcribed spacer (ITS-2) of nuclear ribosomal DNA was amplified from the genomic DNA sample by the PCR and sequenced directly. The sequence obtained was identical to the ITS-2 sequence published previously for *H. contortus *(GenBank accession number X78803; [24]). Subsequently, the complete mt genome was amplified by long-PCR from the genomic DNA in two overlapping regions (~5 kb and ~10 kb, respectively) [14]. Each amplicon appeared as one abundant band of the appropriate size on an agarose gel. Short tags (300–400 bp) were sequenced from the 10 kb and 5 kb amplicons (within the *cox1 *and *rrnL *genes, respectively) to verify their specificity and identity. Following DNA quantitation for each amplicon, the two amplicons (5 μg from each) were pooled and sequenced directly in a single reaction using 454 technology (whole genome sequencing protocol).

### Validation of sequencing via 454 technology by comparison with data mined from public databases

The recently developed 454 technology platform [18] has been utilized for the sequencing of the complete nuclear genomes from a range of organisms [22,25-28]. Although, prior to the present study, it had not been applied to mt genomes of nematodes, a study of marine microbes [22] has shown this technology (together with the Sanger method) to be particularly suited for sequencing AT-rich regions with complex secondary structures, yielding higher quality sequences and costing less overall to carry out than Sanger sequencing alone. This information indicated that 454 technology was perfectly suited to the AT-rich mt genomes of strongylid nematodes, but this required verification.

**Figure 1:**
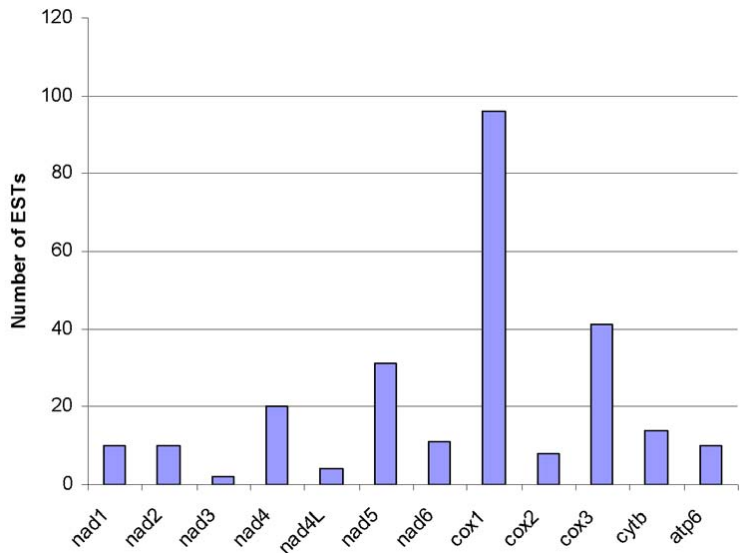
Histogram showing the numbers of ESTs mined from public databases (see [30, 31]) and representing each of the 12 protein coding mitochondrial genes (x-axis) from *Haemonchus contortus*.

The mt genome sequence (designated HcMG-454; accession number EU346694) of *H. contortus *determined using 454 technology was automatically assembled into one contig of 14,055 bp. This sequence was then compared with extensive mt gene sequences available in public databases. For this comparison, ESTs (n = 24,014) from 12 different cDNA libraries, constructed from various lifecycle stages or tissues of *H. contortus *[29] available *via *[30] (n = 10,000 ESTs) and [31] (n = 14,014 ESTs) were mined for mt gene data. In total, 257 ESTs were obtained (Table 1) and found to have high similarity (90–100% at the nucleotide level) to the sequences of protein coding genes of the *H. contortus *in the mitochondrial genome determined in the present study. An examination of the number of ESTs obtained for each protein coding mitochondrial gene (Figure 1) revealed that *cox1*, is much more highly represented than any other gene (96 ESTs). The *cox3*, *nad5 *and *nad4 *genes are also highly represented (41, 31 and 20 ESTs, respectively). Given the number of EST reads contained within these libraries (~24,000) and the random nature in which EST reads are generated (randomly selected cDNA clones), it seems reasonable to infer that the relative proportion of the currently available EST contigs for *H. contortus *which match each mitochondrial gene are related to the relative abundance of the mRNA transcripts for each gene *in vitro*.

**Table 1:** Summary of *Haemonchus contortus *expressed sequence tags (ESTs) mined from public databases ([30, 31]) using the full length sequence of each of the 12 protein coding genes from the mitochondrial genome obtained *via *454 technology in the present study.

Gene	Gene Length (bp)	ESTs	Read Length (bp)	Identity^a^
*nad1*	879	10	118–586	95–99%
*nad2*	846	10	266–437	92–99%
*nad3*	336	2	193–229	97–98%
*nad4*	1230	20	203–563	93–98%
*nad4L*	234	4	105–110	91–97%
*nad5*	1582	31	237–597	96–99%
*nad6*	447	11	124–347	90–100%
*cox1*	1576	96	166–791	94–100%
*cox2*	693	8	293–663	96–100%
*cox3*	766	41	221–604	94–97%
*cytb*	1113	14	157–596	94–99%
*atp6*	600	10	266–429	92–97%

^a^Based on unannotated (raw) EST data from public databases

In addition to mining EST data from online databases, GSS data (available *via *[30]) were mined for mt gene sequences using an unpublished *cox1 *sequence as an *in silico*-bait. We discovered contigs 002363 (14,442 bp) and 002480 (14,814) which were then mapped against HcMG-454 at both the nucleotide and amino acid levels (for protein coding genes), achieving complete coverage with > 99.7% similarity at the nucleotide level. Of the nucleotide differences, 6 were found within protein coding genes. Four of these single nucleotide differences between HcMG-454 and contigs 002363 and 002480 were interpreted to represent intraspecific variation in the nucleotide sequence of the mt genome of this species, as none resulted in an amino acid change. Two appeared to be sequencing errors, as they resulted in frameshifts in the inferred amino acid sequence: both were found in the cox1 gene and were base-called using alignments against available EST data (see Materials and methods section). All other nucleotide alterations (n = 34) were within non-coding regions.

### Characteristics of the mt genome of *Haemonchus *contortus and comparative analysis with those from related nematodes

**Figure 2:**
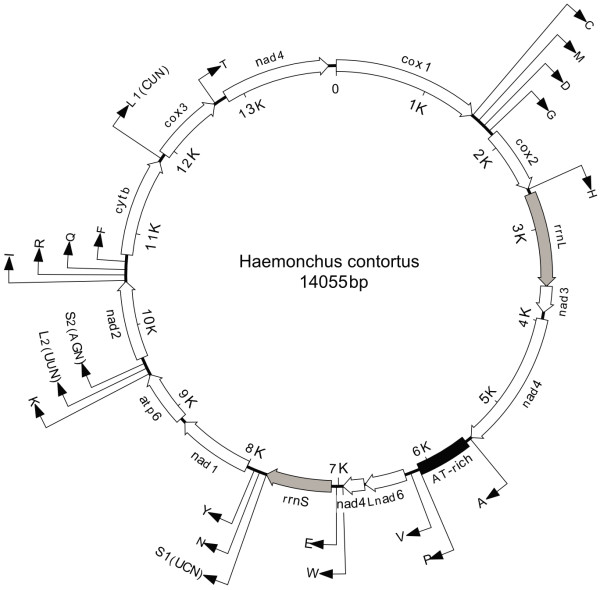
Complete circular mitochondrial genome map for *Haemonchus contortus*. All 12 protein coding genes and the large and small mitochondrial ribosomal subunit genes indicated in italics. All *trn *genes indicated by their corresponding single letter amino acid code. Distinctions between the two Leucine *trn *genes and between the two Serine *trn *genes are indicated by the corresponding anticodon for each *trn *(in brackets). Direct of transcription is indicated by arrow. Diagram is presented to scale.

The complete mt genome sequence of *Haemonchus contortus*, HcMG-454, was characterized (Figure 2). It was 14,055 bp in length and was assembled as a single contig from 5,965 overlapping sequence reads within the 454 sequencing platform. The mt genome of this species is ~300–400 bp larger than those characterised for related species (Strongylida), which range from 13.6–13.7 kb [32,33], but is within the range of previously published mt genomes for chromadorean nematodes (~13.6–14.3 kb) [32,34-37]. The larger size relates primarily to an apparent expansion of the AT-rich control (465 bp *versus *268, 173 and 304 bp for the corresponding region in *Ancylostoma duodenale*, *Necator americanus *and *Cooperia oncophora*, respectively) as well as the presence of slightly longer non-coding regions between many of the transfer RNA genes. The mt genome sequence of *H. contortus *is AT-rich (78.1%) and exhibits an asymmetrical nucleotide usage: 33.2% for A, 44.9% for T, 15.5% for G and 6.5% for C in the coding strand (Table 2). Within the Strongylida, AT-richness in the mt genome has been found to range from 76.6–77.2% [32,33], and, as is observed here for *H. contortus*, T is the most dominant nucleotide in the coding strand (47.1–49.2%) [32,33]. Given the AT bias (~78%) in the mt genome of *H. contortus*, there is a considerable bias in codon usage. Consequently, ATA (Methionine/translation initiation), ATT (Isoleucine), TTA (Leucine), and TTT (Phenylalanine) were the most commonly used codons (5.6, 6.9, 8.2 and 11.0%, respectively), compared with those containing numerous G and/or C residues, such as CGC (Glycine), GCC (Glycine) and GCG (Glycine) (0.0, 0.1 and 0.5%, respectively). These findings are consistent with previously characterized nematode mt genomes [32,34-37].

**Table 2:** Nucleotide composition (%) for all characterised mitochondrial genomes from species of Strongylida.

Species	A	G	C	T	A+T	Reference
*Haemonchus contortus*	33.2	15.5	6.5	44.9	78.1	Present study
*Ancylostoma duodenale*	28.3	16.7	6.6	48.4	76.7	[32]
*Necator americanus*	27.4	17.1	6.3	49.2	76.6	[32]
*Cooperia oncophora*	30.1	16.3	6.5	47.1	77.2	[33]

**Figure 3:**
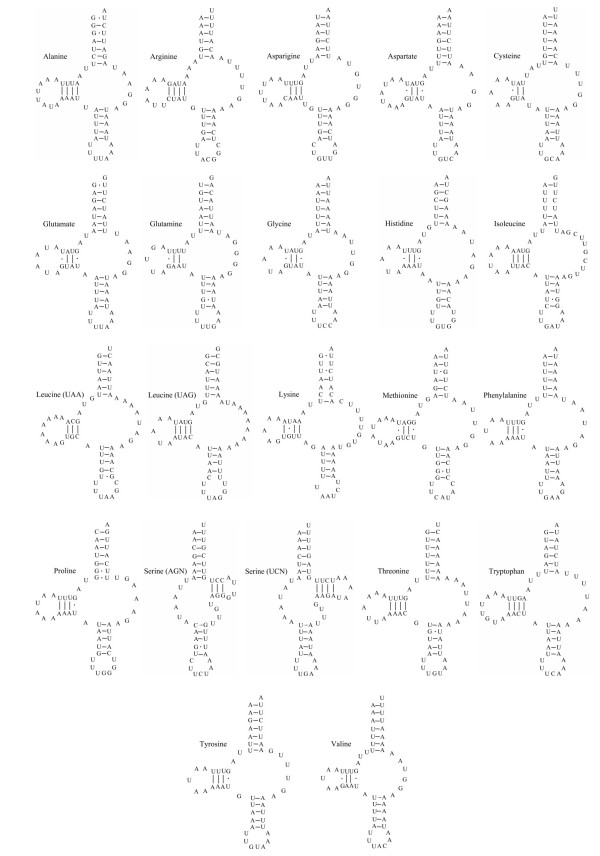
Secondary structures predicted for the 22 *trn *genes in the mitochondrial genome of *Haemonchus contortus*.

**Figure 4:**
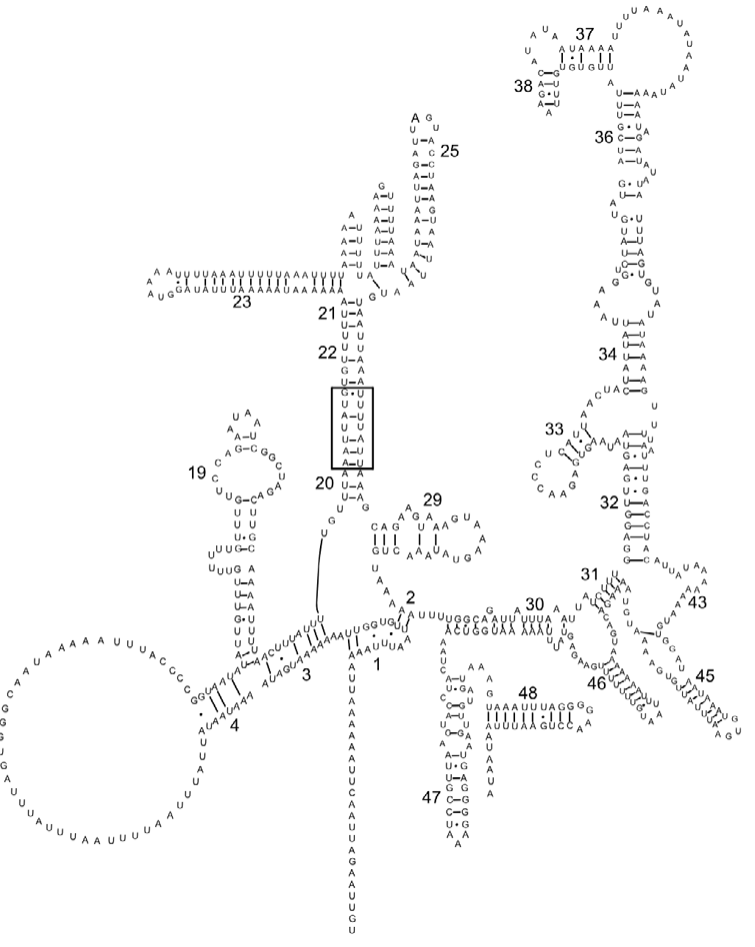
The inferred secondary structure of the mitochondrial small ribosomal subunit (*rrnS*) for *Haemonchus contortus*. Bonds between C:G and U:A nucleotides indicated by a straight line; bonds between U:G indicated by a closed circle and between A:G indicated by an open circle as per Hu et al [32]. Conserved secondary structure elements defined by Dams et al. [40] indicated by numbers 1–48. Insert of 7 nucleotides indicated in box.

**Figure 5:**
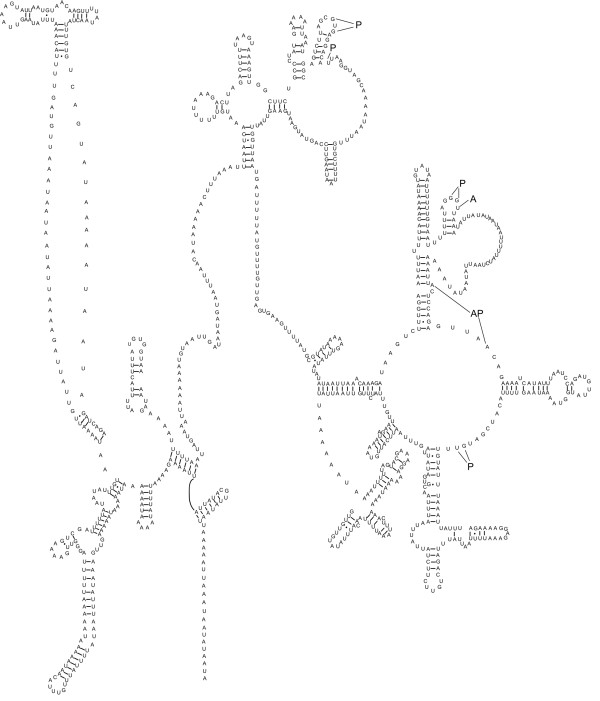
The inferred secondary structure of the mitochondrial large ribosomal subunit (*rrnL*) for *Haemonchus contortus*. Bonds between C:G and U:A nucleotides indicated by a straight line; bonds between U:G indicated by a closed circle and between A:G indicated by an open circle as per Hu et al [32]. Binding sites for the amino-acyl trn (A), peptidyl-transferase (P) or both (AP) as defined by Noller et al. [41] indicated in bold text according to Hu et al. [32].

The gene content of the mt genome of *H. contortus *is consistent with that reported for other chromadorean nematodes [32,34-37] in having 12 protein coding genes (the cytochrome *c *oxidase subunits 1–3 (*cox1-cox3*), the nicotinamide dehydrogenase subunits 1–6 (*nad1-nad6 *and *nad4L*), cytochrome *b *(*cytb*) and adenosine triphosphatase subunit 6 (*atp6*)), 22 transfer RNA (tRNA) genes (Figure 3) and the small (*rrnS*) (Figure 4) and large (*rrnL*) ribosomal subunits (Figure 5). As for other chromadorean nematodes studied to date [4], no adenosine triphosphatase subunit 8 (*atp*8) gene is present, and all genes are predicted to be transcribed from the same strand and in the same direction. Examination of the overall genome structure revealed that the *H. contortus *mt genome has gene arrangement GA2 (see Figure 2 in ref. [32]) which is consistent with all previously published mt genomes for the Strongylida [32,33] and some Rhabditida (including *Steinernema carpocapsae *[38] and *Caenorhabditis elegans *[39]).

Although the mt genome of *H. contortus *is highly AT-rich, this richness is not distributed evenly (Table 3). Like other chromadorean mt genomes characterised to date [32,34-37], the mt genome of *H. contortus *contains an AT-rich (predicted control) region which, as the name denotes, has the highest content of A and T nucleotides (89.4%) for any region in this genome. The *rrnL *and *rrnS *genes are also highly AT-rich (83.4% and (81.4%, respectively), consistent with other nematodes [32,34-37]. The protein coding genes with the highest AT content are *nad2-4 *and *nad4L*, in accordance with the species of Strongylida characterised to date [32,33]. The protein coding genes with the lowest AT content are *nad1 *(69.7%) and *cox1 *(70.8%). Interestingly, although *cox1 *is the gene with the lowest AT content in known mt genomes of the Strongylida, *nad1 *appears to have a lower AT content (69.7%) in *H. contortus *relative to *An. duodenale*, *N. americanus *and *Co. oncophora *(74.1%, 74.0% and 72.0%, respectively). In the other Strongylida, the gene with the next lowest AT-richness is *cox2 *(70.8–72.0%).

**Table 3:** AT contents (%) of the 12 protein coding genes, large and small ribosomal RNA subunits and AT-rich regions for all Strongylida for which complete mitochondrial genome data are currently available

Gene	*Haemonchus contortus*	*Ancylostoma duodenale*	*Necator americanus*	*Cooperia oncophora*
*atp6*	76.8	77.8	75.9	76.6
*cox1*	70.8	69.3	69.5	70.8
*cox2*	73.6	70.8	72.0	72.0
*cox3*	73.1	74.3	73.9	72.7
*cytb*	74.7	74.0	74.3	73.4
*nad1*	69.7	74.1	74.0	72.2
*nad2*	83.3	81.2	80.5	81.0
*nad3*	81.1	78.3	79.2	79.8
*nad4*	79.5	78.5	79.0	79.5
*nad4L*	82.2	80.3	80.3	80.7
*nad5*	77.5	77.1	79.3	78.6
*nad6*	75.6	79.3	77.7	79.2
*rrnS*	81.4	76.6	75.3	75.6
*rrnL*	83.4	81.1	81.4	82.2
AT-rich	89.4	90.1	83.2	85.2
Whole genome	78.1	76.7	76.6	77.2

Previous studies of the mt genomes from the Strongylida [32,33] have indicated high variation in the translation initiation and termination codons among the protein coding genes within the mt genome of a single species (Table 4). Within the hookworms, an "ATT" initiation codon is most common (9 and 7 of all 12 protein coding mt genes for *An. duodenale *and *N. americanus*, respectively). Both of these hookworms also use a "TTG" initiation codon for selected genes. Also, *An. duodenale *uses an "ATG" initiation codon for *nad6 *and *N. americanus *uses an "ATA" initiation codon for *cox2*. The initiation codons used by *Co. oncophora *also appear to be relatively heterogeneous (seven "ATT", two "ATA", one "TTG", one "TTA" and one "GTA"). This is also true for *H. contortus *with initiation codons ranging from ATT (4 genes), ATA (4 genes), TTG (2 genes), GTT (1 gene) and AAT (1 gene). The most commonly used termination codon for the *H. contortus *mt genes is TAA (7 genes), however, TGT (2 genes), TG (1 gene), TAG (1 gene) and TT (1 gene) are used also.

**Table 4:** Comparative analysis of the transcription initiation and termination codons utilized by the protein coding genes in the mitochondrial genomes of all species of Strongylida for which data are currently available.

Protein	*Haemonchus contortus*	*Ancylostoma duodenale*	*Necator americanus*	*Cooperia oncophora*
ATP6	ATT/TAA	ATT/TA	ATT/T	GTA/TAA
COX1	AAT/TT	ATT/TA	ATT/TAG	ATT/TAA
COX2	ATA/TAG	ATT/TAA	ATA/TAA	ATT/TAA
COX3	ATA/TG	ATT/T	ATT/T	TTG/TAT
CYTB	ATT/TAA	ATT/TA	ATT/TAG	ATA/TAT
NAD1	ATA/TAA	ATT/TAA	TTG/TA	ATT/TTT
NAD2	TTG/TAA	TTG/TAA	ATT/TAG	ATA/TTT
NAD3	TTG/TAA	ATT/TAG	TTG/TAA	ATT/TTT
NAD4	ATA/TAA	TTG/TAA	TTG/TAA	ATT/TAA
NAD4L	ATT/TGT	ATT/TAA	ATT/TAG	TTA/TAG
NAD5	ATT/TGT	ATT/T	ATT/T	ATT/TAT
NAD6	GTT/TAA	ATG/TAG	TTG/TAA	ATT/TAA

Pairwise comparisons of amino acid sequences inferred from coding genes for *H. contortus *were made with the corresponding sequences from each of the three previously characterised Strongylida (*An. duodenale*, *N. americanus *and *Co. oncophora*) (see Table 5). The overall amino acid sequence similarity ranged from 51.8–95.1%. The predicted proteins with the greatest similarity were COX1 (94.7–95.1%), COX3 (90.8–92.5%) and COX2 (87.1–91.0%). The proteins with the least similarity were NAD6 (54.5–55.2%) and NAD2 (51.8–56.1%). The ranking from least to most variable is: COX1, COX3, COX2, ATP6, CYTB, NAD1, NAD3, NAD4, NAD4L, NAD5, NAD6 and NAD2. In addition to encoding the most conserved protein, the *cox1 *gene is also the least AT-rich, suggesting that it is an attractive target for the design of relatively conserved primers, which may also apply to the genes *cox2 *and *cox3*. Interestingly, six of the 12 peptides (ATP6, CYTB, NAD1, NAD3, NAD4L and NAD6) inferred from the mt genome sequence of *H. contortus *(Trichostrongylidae) are more similar in sequence to those of the two hookworms (Ancylostomatidae), *An. duodenale *and *N. americanus*, than for *Co. oncophora *(Trichostrongylidae). Unlike the mt genome sequences characterised for *An. duodenale *[32], *N. americanus *[32] and *H. contortus *(present study), which were obtained from individual specimens, the mt genome of *Co. oncophora *was sequenced from pooled larvae [33]. An examination of the nucleotide sequence provided for this species reveals a range of single nucleotide polymorphisms (SNPs) scattered throughout various genes [33]. It is likely that these SNPs have contributed to ambiguity in the translated amino acid sequence used and examined in the present study, and may explain the discrepancy between the amino acid sequence similarities and the current taxonomic relationships of these strongylid species. Also, because the mt genome of *Co. oncophora *was determined from pooled larvae rather than single worms, there is some potential that the sample sequenced contained one or more heterologous species of strongylid. Therefore, to prevent any sequence ambiguity, it is recommended that single, morphologically identified adult male nematodes are used for mt genome sequencing.

**Table 5:** Pairwise comparison (%) of the amino acid sequence inferred for each of the 12 protein coding mitochondrial genes from *Haemonchus contortus *(Hc) *versus *all previously described protein coding mt genes from *Ancylostoma duodenale *(Ad), *Necator americanus *(Na) and *Cooperia oncophora *(Co).

Protein	Hc/Ad	Hc/Na	Hc/Co
ATP6	83.1	82.5	80.4
COX1	94.7	95.1	95.1
COX2	87.1	89.5	91.0
COX3	92.5	90.8	92.9
CYTB	81.9	80.3	79.7
NAD1	75.4	74.6	72.5
NAD2	51.8	53.6	56.1
NAD3	73.6	77.4	66.0
NAD4	73.1	70.4	72.9
NAD4L	72.1	75.0	69.1
NAD5	71.4	70.7	71.8
NAD6	55.2	54.5	54.5

All circular nematode mt genomes described to date have been found to contain 22 *trn *genes [32,34-37], and *H. contortus *is no exception (Figure 3). The *trn *genes for this species range from 53–61 bp, which is consistent with other nematodes [32,34-37]. The secondary structure of the *trn*S genes consists of a 7–8 bp amino-acyl arm and a 5 bp anticodon stem, with a T/U residue always preceding, and a purine always following the anticodon. Twenty of the 22 *trn*S genes were found to have a 3–4 bp DHU arm with a 5–8 bp DHU loop and a 6–11 bp TV-replacement loop instead of a TψC arm. The two exceptions to this are the two serine (AGN and UCN) *trn*S genes which have a 6 bp DHU replacement loop instead of the DHU arm, and a 3–4 bp TψC arm with a 4–5 bp TψC loop instead of the TV replacement loop. These findings are consistent with the *trn*S genes described for all 11 chromadorean nematodes characterised to date [32,34-37].

The *rrnS *and *rrnL *genes of *H. contortus *were consistent in length (702 and 915 bp, respectively) with those reported previously for most nematodes [32,34-37], showing 77–78% and 75–76% sequence similarity, respectively, with homologous sequences from *An. duodenale*, *N. americanus *and *Co. oncophora*. The secondary structures for *rrnS *(Figure 4) and *rrnL *(Figure 5) were inferred by mapping against the appropriate secondary structures for *N. americanus *[32], originally based on *Escherichia coli *models [40] and predicting, either manually or through computer assisted predictions (MFOLD), changes caused by various mutations.

In accordance with the two known hookworm mt genomes [32], the *rrnS *structure consists of four relatively conserved domains (A-D, Figure 4) bound by numerous conserved helices [40]. A 7 nt insert between positions 187–195, apparently interacts with a 7 nt tract between positions 305–311 (see Figure 4) and is predicted to result in a slight alteration of the secondary structure between conserved elements 20 and 25 [40], resulting in an extension of the stem between elements 20 and 22 and the formation of a 4 bp and a 7 bp stem loop between elements 23 and 25 (see Figure 4). Although the secondary structure of the *rrnS *of *Co. oncophora *was not characterised previously [33], the sequence did not reveal the 7 nt tract identified in *H. contortus*. Whether this inferred alteration in the *rrnS *structure in *H. contortus *is unique to this and/or to closely related species remains to be elucidated.

The *rrnL *secondary structure predicted for *H. contortus *is consistent with those predicted for *An. duodenale *and *N. americanus *[32] and consists of four major stem-loop domains (1–4) (Figure 5) which appear to be conserved [32,34-37]. The amino-acyl *trn *binding sites (A) and peptidyl-transferase sites (P), first described from *Escherichia coli *by Noller *et al*. [41] and later recognized in nematodes [32], are present. Hu *et al*. [32] found that, although these "A" and "P" binding sites were present in hookworms, the exit site (E) proposed by Noller *et al*. [41] was not found in the *rrnL *of the hookworms or *Ascaris *sp., *Ca. elegans*, *Onchocerca volvulus *or *Trichinella spiralis *[39,42,43]. Also, no E site was found within the secondary structure of the *rrnL *of *H. contortus*, demonstrating consistency with previous findings and lending further support to the hypothesis that many nematode *rrnL *genes do not have a recognizable exit site [32] or, at least, not one comparable with that of *E. coli *[41]. Although the secondary structure of the *rrnL *of *Co. oncophora *has not been reported previously [33], the sequence similarity (75–79%) among the *rrnL *genes of *H. contortus*, *An. duodenale *and *N. americanus *suggests a conserved secondary structure for these Strongylida.

**Figure 6:**
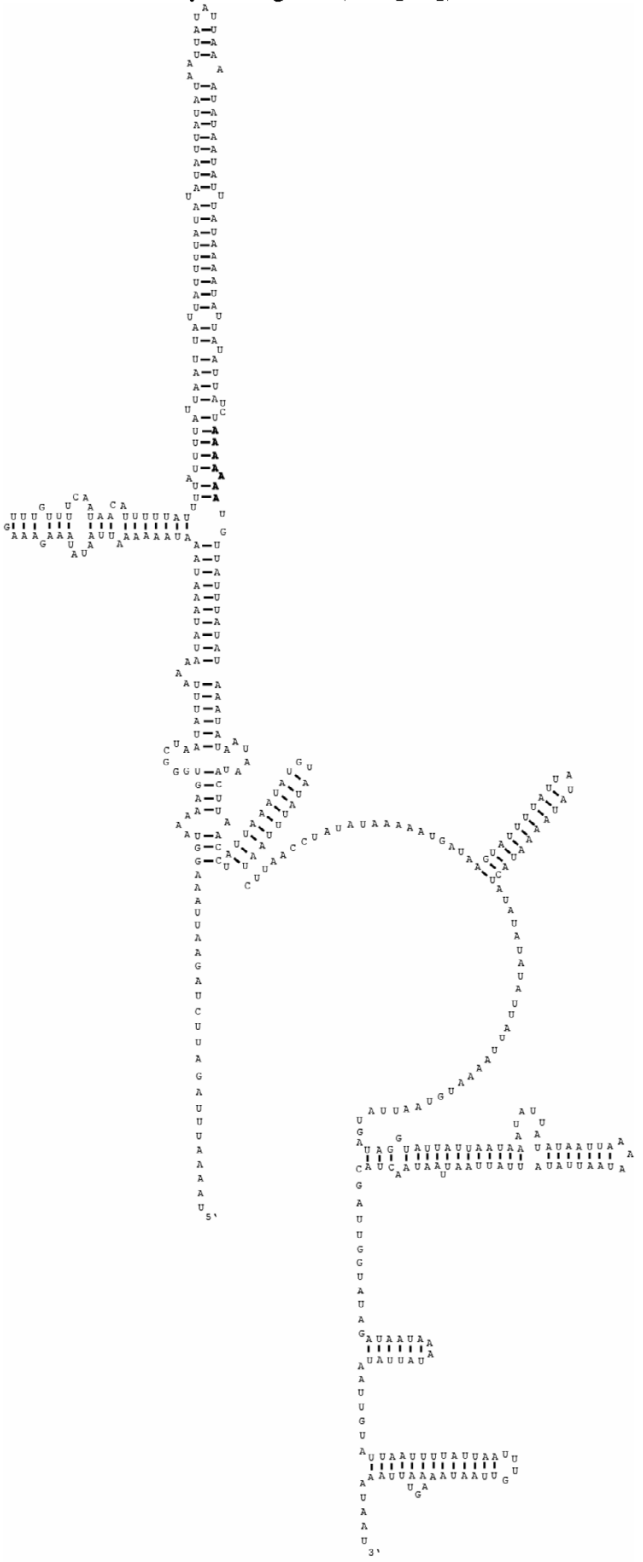
Secondary structure predicted for the AT-rich region in *Haemonchus contortus*. Poly-A region (see [32].) indicated in bold type.

The 36 genes of the *H. contortus *mt genome comprise ~94% of the entire mt genome. The remaining ~6% consists of non-coding regions, which are predicted to be functionally involved in the regulation of transcription, translation and/or replication [3,4,32]. The largest of these non-coding regions is the AT-rich region, which is 465 bp in length (Figure 6). As for other nematodes with the gene arrangement GA2 [3,4,35], this region was located between the *nad5 *and *nad6 *genes. Also, like other GA2 nematodes, the AT-rich region in *H. contortus *is flanked (5') by the *trnA *gene and (3') by the genes *trnP *and *trnV*, and a short (~35 bp) non-coding region between genes *trnP *and *trnV*. This region is 157–292 bp longer than the corresponding region reported for *An. duodenale *(278 bp), *N. americanus *(173 bp) [32] and *Co. oncophora *(308 bp) [33]. However, as described by Hu *et al*. [3], the AT-rich region is likely to exhibit significant intraspecific length polymorphism. As for hookworms [32], in *H. contortus*, this region exhibits a complex stem-loop formation (Figure 6), hypothesized to be involved in mt replication in the mt genome [32]. In addition to having complex secondary structure, the AT-rich region can contain repetitive elements. The AT-rich region of *Ca. elegans *contains six repeats (CR1-CR6) of an identical 43 nucleotide sequence [39]. Although such a repetitive element was not found in the mt genomes of *An. duodenale *or *N. americanus *[32], regions believed to represent remnants of these repeats have been found in the mt genome of *Ascaris *sp., in the same study as that of *Ca. elegans *[39]. In the present study, the AT-rich region of *H. contortus *does not contain the repetitive element described for *Ca. elegans*. However, a sequence alignment of the AT-rich regions from these two species did indicate that some of the "TAT" portions [32] of the repetitive elements described for *Ca. elegans *may be present. Lastly, Hu *et al*. [32] described a poly-A (n = 7–8) region within the AT-rich region of all chromadorean nematodes characterised at the time. A similar poly-A region is present in the AT-rich region of *H. contortus *(see Figure 6). It has been hypothesized [32] that such poly-A region are involved in mt gene replication, as proposed for poly-T regions found in the control region for some vertebrate and insect species [44-46]. This proposal warrants experimental investigation.

Another non-coding region between *cox1 *and *nad4 *has been described from all previously characterized nematodes exhibiting gene arrangement GA2 [32,39], namely *An. duodenale*, *N. americanus*, *As. suum*, *S. carpocapsae *and *Ca. elegans*. In the latter three nematodes, this region forms a stem-loop structure [39]. However, the region does not appear to form a stem loop structure in *An. duodenale *nor in *N. americanus *[32], and does not appear to form a stem loop in the *H. contortus *mt genome reported herein. In addition, nematodes with gene arrangement GA3, presently represented by the filarial nematodes (*B. malayi*; accession no. AF538716), *O. volvulus *[42] and *D. immitis *[36]) from which complete mt genomes have been described, also have a gene order juxtaposed such that the *cox1 *gene follows the 3' end of *nad4*. There is no non-coding region between these two genes. This information suggests that if secondary structure in the non-coding region between *cox1 *and *nad4 *is involved in regulating replication, transcription and/or translation, it is not a universal requirement for all nematodes. Hu *et al*. [32] found a third non-coding region between the genes *nad3 *and *nad5 *(80 bp and 55 bp for *An. duodenale *and *Ne. americanus*, respectively). A similar region is present in *H. contortus*. In *An. duodenale*, this region was inferred to form a 6 bp and a 9 bp stem-loop [32], and it is proposed to be involved in interactions with RNA processing enzymes, as has been reported for similar stem-loop structures in humans [47]. No such secondary structure was found for *Ne. americanus *[32], nor was it predicted here for the corresponding region in *H. contortus*. The degree to which such structures are involved in regulating molecular events in the mitochondria of nematodes in not yet clear and is an exciting area for future research.

### 454 technology as a high-throughput method for sequencing mitochondrial genomes

The present study demonstrates the utility of the 454 technology platform for the sequencing of AT-rich mt genomes combined with mapping against a scaffold of available sequences mined from public EST and/or GSS databases. This approach represents a "scaled-down" version used for sequencing complete nuclear genomes [16,18,20,22,48] and overcomes the significant limitations of sequencing AT-rich templates using conventional approaches [14]. Presently, the equipment costs associated with this platform are likely to be prohibitive for most laboratories. Consequently, most laboratories will likely utilise commercial services if employing this method. In terms of cost, sequencing of a small number of mt genomes using this platform is probably not directly competitive with conventional Sanger sequencing *via *primer walking and/or cloning. However, if one considers the vast improvements, in terms of efficiency, in relation to the complete sequencing and sequence assembly of these genomes, we contend that this approach is a practical alternative.

The period from genomic DNA extraction to the final output of a complete, assembled mt genome takes ~2–3 weeks. In research applications, where a panel of conserved primers is available for primer walking, this level of output is probably achievable. However, for applications where such primers are not available (because of a lack or absence of sequence data from the organism or a related species) and must be designed *de novo *based on sequencing results, this present approach has major advantages. Equally, shotgun cloning-based sequencing would not be as efficient, unless a high-throughput (e.g., robotic) system were available for the rapid isolation of large numbers of clones for plasmid purification and subsequent sequencing to ensure adequate coverage of the mt genome, thus substantially increasing costs. Furthermore, by either a primer walking or shotgun cloning approach, a significant amount of bioinformatic processing is required for contig assembly following sequencing, which is not required using the 454 platform because contig assembly is automated. Economically, if 454 technology is applied as a high-throughput system in which multiple mt genomes are sequenced simultaneously, the direct costs per such genome (~USD 1,250) becomes directly comparable with, if not less expensive than other approaches. The benefits in terms of efficiency presented by this technology are considered substantial.

In addition to cost and efficiency benefits, the 454 sequencing method may provide greater reliability in the sequence output (estimated sequencing error in the present study was 2 errors in 14,055 bp of sequence) which will likely improve as newer versions (e.g., the GS-FLX system) of the sequencing platform and assembly software are made available. Given the cost and laborious nature of primer walking and/or conventional cloning-based methods, most mt genomes presently available have been assembled as a single contig following uni- or bi-directional sequencing, resulting in a one- to two-fold coverage of the genome (forward and reverse strands). The sequencing of large numbers of overlapping sequences generated from a template using the 454 technology offers a substantial increase in coverage. In the present study, the complete mt genome was assembled from ~6,000 overlapping sequences, each read being ~100 bp [18]. This translates into a total sequence output from one reaction of ~600,000 bp, resulting in substantial "coverage" of the mitochondrial genome, which would be impractical, too laborious and costly to achieve using conventional sequencing approaches.

Previously, it has been suggested that the 454 technology may be less reliable for sequencing homopolymeric and repetitive elements [18,22], which have been detected previously in the mt genomes of nematodes, particularly in the AT-rich region [38]. In the mt genome of *H. contortus *determined, 66 such regions (of 7–9 As or Ts) were identified, but there was no evidence (based on comparison with EST and GSS datasets) of any problems herein with sequencing through such elements. In the present study, the discovery of a complete mt genome sequence for *H. contortus *in a GSS database allowed the direct evaluation of the 454 sequencing output, and did not reveal any sequencing errors. As homopolymeric and repetitive elements can occur in the AT-rich region and elsewhere, it may be warranted, for organisms for which no prior sequence data are available, to undertake conventional Sanger sequencing to independently verify the accuracy of such sequence elements determined by 454 technology.

## Conclusion

The present study demonstrates clearly the utility and practicality of 454 technology for the sequencing of mt genomes. The high-throughput capacity of this approach provides unique prospects for large-scale mt sequencing projects as a foundation for population genetic, evolutionary and ecological studies [4]. The present investigation also discovered substantial amounts of mt data present in EST and GSS data sets for *H. contortus*, suggesting that databases available for other species will provide a useful resource for the mining of data to assist in the annotation, assembly and analyses of mt genome sequence data.

The sequence (HcMG-454) reported in this paper is available in the GenBank database under accession number EU346694.

## Methods

### Production of *Haemonchus *contortus, isolation of genomic DNA and verification of specific identity by molecular means

Adults of *H. contortus *(McMaster strain) were produced in a helminth-free sheep [49]. Adult worms were isolated from the abomasum (= stomach) and washed extensively in physiological saline (25°C). Using a dissecting microscope (5× magnification), the sex of individual worms was verified microscopically, and male and female worms separated. Individual worms were transferred to sterile, screw-top cryogenic tubes (Nunc) and frozen (-70°C) in a minimal amount of buffer. After thawing, total genomic DNA was isolated from an individual male of *H. contortus *using a standard sodium dodecyl-sulphate/proteinase K treatment [50], followed by purification over a mini-column (Wizard, Promega). The specific identity of the specimen was verified by PCR-based amplification of the second internal transcribed spacer (ITS-2) of nuclear ribosomal DNA using an established method, followed by mini-column purification of the amplicon and subsequent automated sequencing (BigDye chemistry v3.1) [51]; the ITS-2 sequence determined was identical to that with GenBank accession no. X78803 [52].

### Long-PCR amplification of two mt genome regions

The complete mt genome was amplified from ~10% (20–40 ng) of the genomic DNA from the individual specimen by long-PCR (BD Advantage 2; BD Biosciences) as two overlapping amplicons (large and small), using the protocol described by Hu *et al*. [14] with minor modifications. The large (~10 kb) amplicon was produced using the primers MH43F (forward: 5'-TTCTTATGAGATTGCTTTTTCT-3') and MH40R (reverse: 5'-GAATTAAACTAATATCACGT-3'). Briefly, the PCR (50 μl) was conducted using 10 pmol of each of the two oligonucleotide primers, 100 μM of each dNTP, 3 mM MgCl_2 _and 1 U of BD Advantage 2 *Taq *polymerase (BD Biosciences) using an ABI 2720 thermal cycler (ABI), employing the following cycling protocol: one cycle at 94°C for 2 min (initial denaturation), followed by 35 cycles of 94°C for 30 s (denaturation), 50°C for 30 s (annealing) and 65°C for 10 min (extension), followed by a final extension at 65°C for 10 min (utilizing the appropriate positive and negative controls). The small (~5 kb) amplicon was generated using the primer set MH39F (forward: 5'-TAAATGGCAGTCTTAGCGTGA-3') and MH38R (reverse: 5'-TAAATGGCAGTCTTAGCGTGA-3') under the same conditions, with the exception that the extension temperature was reduced to 60°C [14]. The specificity of the two primer sets had been validated previously [14]. Following the PCR, each amplicon was subjected to electrophoresis in 1% agarose, using a 1 kb DNA Ladder (Promega) to estimate size. Amplicons were then purified over a mini-column (Wizard, Promega) and quantified spectrophotometrically using a NanoDrop ND-1000 UV-VIS spectrophotometer v.3.2.1 (NanoDrop Technologies). The specificity of the PCR conditions and amplicons was verified by partial, automated Sanger sequencing (employing BigDye Chemistry v3.1), using primers COIF (5'-TTTTTTGGGCATCCTGAGGTTTAT-3') and MH28R (5'-CTAACTACATAATAAGTATCATG-3') (large fragment) and MH37F (5'-GGAGTAAAGTTGTATTTAAAC-3') and MH40R (5'-GAATTAAACTAATATCACGT-3') (small fragment) [3,35].

### Automated sequencing using 454 technology

The two amplicons (~5 kb and 10 kb; 5 μg of each) spanning the mt genome of *H. contortus *were pooled and subsequently sequenced using the Genome Sequencer 20 (GS 20; Roche) according to the protocol provided [18]. The mt genome sequence (designated HcMG-454; GenBank accession no. EU346694) was assembled automatically and compared against EST and GSS sequences for *H. contortus *available from public databases [30,31]. HcMG-454 was scanned for open reading frames (ORFs) using ORFinder [53], employing the "Invertebrate Mitochondrial" option. Protein coding genes were identified by BLASTx analysis of the inferred amino acid sequences, and the initiation and termination codons identified by alignment at the nucleotide (ClustalX) and amino acid (Clustal W) levels against the mostly closely related nematode species for which the mt genome has been characterized. The positions and secondary structures of all transfer RNA (*trn*) genes were identified or determined using tRNAscan SE 1.21 [54] using the "Nematode Mito" source option and the "Invertebrate Mito" tRNA isotype prediction option. The *rrnL *and *rrnS *genes and AT-rich control region were identified by BLASTn analysis and comparisons with respective sequences within the mt genomes of *An. duodenale*, *Ne. americanus*, *Co. oncophora *and *Ce. elegans *(see GenBank accession numbers AJ417718, AJ417719, AY265417 and X54252, respectively). The secondary structures for the *rrnL *and *rrnS *were predicted by sequence alignment against *rrnL *and *rrnS *from *Ne. americanus *[32] with secondary structures for variable regions determined using MFOLD [55]. The secondary structure of the AT-rich region was also determined using MFOLD. Following genome annotation, each protein-coding gene was conceptually translated using Translation Tool (v.3.1) [56] using the Invertebrate Mitochondrial Code" option. Amino acid sequences were aligned with those inferred from previously characterised mt genomes for species of Strongylida (i.e., *An. duodenale*, *Ne. americanus *and *Co. oncophora*) and *C. elegans *using Clustal W [57], adjusted manually and verified using BioEdit v.7.0.3 [58].

## Authors' contributions

ARJ and RBG conceived and designed the research plan and carried out the project. ARJ and MH analysed the sequence data, and ARJ, MH and RBG interpreted the data. RBG supervised and coordinated the sequencing. ARJ and RBG drafted the manuscript. All authors contributed to the manuscript, and read and approved the final manuscript.
